# CuGenDBv2: an updated database for cucurbit genomics

**DOI:** 10.1093/nar/gkac921

**Published:** 2022-10-22

**Authors:** Jingyin Yu, Shan Wu, Honghe Sun, Xin Wang, Xuemei Tang, Shaogui Guo, Zhonghua Zhang, Sanwen Huang, Yong Xu, Yiqun Weng, Michael Mazourek, Cecilia McGregor, Susanne S Renner, Sandra Branham, Chandrasekar Kousik, W Patrick Wechter, Amnon Levi, Rebecca Grumet, Yi Zheng, Zhangjun Fei

**Affiliations:** Boyce Thompson Institute, Ithaca, NY 14853, USA; Boyce Thompson Institute, Ithaca, NY 14853, USA; Boyce Thompson Institute, Ithaca, NY 14853, USA; Plant Biology Section, School of Integrative Plant Science, Cornell University, Ithaca, NY 14853, USA; College of Horticulture and Forestry Sciences, Huazhong Agricultural University, Wuhan 430070, China; Boyce Thompson Institute, Ithaca, NY 14853, USA; National Watermelon and Melon Improvement Center, Beijing Academy of Agricultural and Forestry Sciences, Key Laboratory of Biology and Genetic Improvement of Horticultural Crops (North China), Beijing Key Laboratory of Vegetable Germplasm Improvement, Beijing 100097, China; Engineering Laboratory of Genetic Improvement of Horticultural Crops of Shandong Province, College of Horticulture, Qingdao Agricultural University, Qingdao 266109, China; Genome Analysis Laboratory of the Ministry of Agriculture, Agricultural Genomics Institute at Shenzhen, Chinese Academy of Agricultural Sciences, Shenzhen, Guangdong 518124, China; National Watermelon and Melon Improvement Center, Beijing Academy of Agricultural and Forestry Sciences, Key Laboratory of Biology and Genetic Improvement of Horticultural Crops (North China), Beijing Key Laboratory of Vegetable Germplasm Improvement, Beijing 100097, China; U.S. Department of Agriculture-Agricultural Research Service, Vegetable Crops Research Unit, Madison, WI 53706, USA; Department of Horticulture, University of Wisconsin, Madison, WI 53706, USA; Plant Breeding and Genetics Section, School of Integrative Plant Science, Cornell University, Ithaca, NY 14853, USA; Department of Horticulture, University of Georgia, Athens, GA 30602, USA; Faculty of Biology, Systematic Botany and Mycology, University of Munich (LMU), 80638 Munich, Germany; Department of Biology, Washington University, Saint Louis, MO 63130, USA; Coastal Research and Educational Center, Clemson University, Charleston, SC 29414, USA; U.S. Department of Agriculture-Agricultural Research Service, U.S. Vegetable Laboratory, 2700 Savannah Highway, Charleston, SC 29414, USA; U.S. Department of Agriculture-Agricultural Research Service, U.S. Vegetable Laboratory, 2700 Savannah Highway, Charleston, SC 29414, USA; U.S. Department of Agriculture-Agricultural Research Service, U.S. Vegetable Laboratory, 2700 Savannah Highway, Charleston, SC 29414, USA; Department of Horticulture, Michigan State University, East Lansing, MI 48824, USA; Beijing Key Laboratory for Agricultural Application and New Technique, College of Plant Science and Technology, Beijing University of Agriculture, Beijing 102206, China; Bioinformatics Center, Beijing University of Agriculture, Beijing 102206, China; Boyce Thompson Institute, Ithaca, NY 14853, USA; U.S. Department of Agriculture-Agricultural Research Service, Robert W. Holley Center for Agriculture and Health, Ithaca, NY 14853, USA

## Abstract

The Cucurbitaceae (cucurbit) family consists of about 1,000 species in 95 genera, including many economically important and popular fruit and vegetable crops. During the past several years, reference genomes have been generated for >20 cucurbit species, and variome and transcriptome profiling data have been rapidly accumulated for cucurbits. To efficiently mine, analyze and disseminate these large-scale datasets, we have developed an updated version of Cucurbit Genomics Database. The updated database, CuGenDBv2 (http://cucurbitgenomics.org/v2), currently hosts 34 reference genomes from 27 cucurbit species/subspecies belonging to 10 different genera. Protein-coding genes from these genomes have been comprehensively annotated by comparing their protein sequences to various public protein and domain databases. A novel ‘Genotype’ module has been implemented to facilitate mining and analysis of the functionally annotated variome data including SNPs and small indels from large-scale genome sequencing projects. An updated ‘Expression’ module has been developed to provide a comprehensive gene expression atlas for cucurbits. Furthermore, synteny blocks between any two and within each of the 34 genomes, representing a total of 595 pair-wise genome comparisons, have been identified and can be explored and visualized in the database.

## INTRODUCTION

The Cucurbitaceae (cucurbit) family consists of about 1000 species in 95 genera, mainly grown in tropical, subtropical and temperate regions around the world (1,2). The family includes numerous important fruit and vegetable crops with high nutrition and flavor values such as cucumber, melon, watermelon, squash, pumpkin etc. In addition, some cucurbits can also be used as containers, musical instruments and sources of oils, and serve as ornaments for festivals, medicines for disorder treatment, as well as model systems for the study of sex determination (3–5). Due to their importance, abundant genetic and genomic resources have been developed for various cucurbit plants during the past 15 years or so, with cucumber representing the first fruit or vegetable crop that had a genome sequence, which was released in 2009 (6).

We have developed the Cucurbit Genomics Database (CuGenDB), which serves as a central portal for cucurbit comparative and functional genomics (7). Since the release of CuGenDB in 2019, thanks to the rapid advances in sequencing technologies, novel or improved reference genomes have been generated for a number of cucurbit species and variety groups. In addition, gene expression profiling data generated using RNA sequencing (RNA-Seq) have been rapidly accumulated for cucurbit species, which have provided broad insights into molecular mechanisms underlying biotic and abiotic stresses, and plant growth and development. Furthermore, high-resolution genomic variants including single nucleotide polymorphisms (SNPs) and small insertions and deletions (indels) have been generated for various cucurbit populations, which have helped to understand the genetic diversity, origin, and domestication of these cucurbit crops, as well as genetic bases of key cucurbit agronomic traits. A platform for efficient distribution, mining and analysis of these newly generated genomic data would benefit the plant research and breeding community. Therefore, an updated CuGenDB to embrace these data and additional associated data mining functions is urgently needed.

To this end, we have developed an updated version of CuGenDB, CuGenDBv2 (http://cucurbitgenomics.org/v2), mainly using Tripal v3.0, which, compared to Tripal v2.0, has substantially improved the efficiency of genomic data loading into the backend PostgreSQL database tables (several hours using Tripal v3.0 versus several weeks per genome using Tripal v2.0) (8). The web interfaces in CuGenDBv2 have been built using the legacy functionalities in Tripal v2.0 (9). In this way, genomic data can be loaded into the PostgreSQL database quickly and the web interfaces customized efficiently. Some modules/functions in CuGenDBv2, such as ‘Expression’, ‘Genotype’ and ‘Synteny Viewer’, have been implemented using Perl/CGI combined with the backend MySQL database.

## DATABASE CONTENTS

### Cucurbit genome assemblies and syntenies

A total of 34 genome assemblies are currently available in CuGenDBv2. These assemblies are from 27 different species or subspecies that belong to 10 genera in the cucurbit family: *Cucumis*, *Citrullus*, *Cucurbita*, *Luffa*, *Momordica*, *Lagenaria*, *Benincasa*, *Sechium*, *Siraitia* and *Trichosanthes* (Table 1). Ten genome assemblies from the genus *Cucumis* are available in CuGenDBv2, including five from cucumber and six from melon. For cucumber, three are from cultivated species *C. sativus* var. *sativus* (Chinese Long, Gy14 and B10), one from the wild progenitor *C. sativus* var. *hardwickii* (PI 183967), and one from the distant wild relative *C. hystrix* (10–13). For melon, five are from different subspecies or variety groups of cultivated species *C. melo* (DHL92, ssp. *agrestis* IVF77, var. *inodorus* Payzawat, var. *reticulatus* Harukei-3 and var. *cantalupensis Charmono*) and one from the wild melon *C. metuliferus* (14–18). For the genus *Citrullus*, six genome assemblies are available, including two from the cultivated watermelon *C. lanatus* ssp. *vulgaris*, an East Asia ecotype 97103 (19) and an America ecotype Charleston Gray (20), one from the possible direct wild progenitor *C. lanatus* ssp. *cordophanus* (21) and one each from the three wild *Citrullus* species, *C. mucosospermus* (USVL531-MDR), *C. amarus* (USVL246-FR2) and *C. colocynthis* (PI 537277). For the genus *Cucurbita*, five genome assemblies are available, with one each from the four cultivated species, *C. maxima* (Rimu), *C. moschata* (Rifu), *C. pepo* (MU-CU-16), *C. argyrosperma* ssp. *argyrosperma* (SMH-JMG-627), and one wild relative, *C. argyrosperma* subsp. *sororia* (22–24). For *Luffa*, two genome assemblies from the cultivated sponge gourd species *L. cylindrica* including that of cultivar P93075, and one from another cultivated species *L. acutangula* (AG-4) are available (25–27). For *Momordica*, two genome assemblies from the cultivated bitter gourd species *M. charantia* (Dali-11 and OHB3-1) and one from a small-fruited wild line, TR, are available (28,29). For *Lagenaria*, one genome assembly from the food-type bottle gourd *L. siceraria* (Hangzhou gourd) and another from the rootstock-type bottle gourd (USVL1VR-Ls) are available (30,31). For the genera *Benincasa*, *Sechium*, *Siraitia*, and *Trichosanthes*, one genome assembly is available for each of the cultivated species in the four genera: *Benincasa hispida* wax gourd (B227), *Sechium edule* chayote, *Siraitia grosvenorii* monk fruit (Qingpiguo) and *Trichosanthes anguina* snake gourd (32–35). Among the 34 genome assemblies currently in CuGenDBv2, four have not been published in literature and are first released in the database, including the cucumber Gy14 genome and genomes of three wild watermelons (*C. mucosospermus* USVL531-MDR, *C. amarus* USVL246-FR2 and *C. colocynthis* PI 537277).

**Table 1.** Cucurbit genome assemblies available in CuGenDBv2

Common name	Latin name	Accession	Version	No. genes	Source
Cucumber	*Cucumis sativus* var. *sativus*	Chinese Long	v3	24 317	(10)
	*Cucumis sativus* var. *sativus*	Gy14	v2.1	22 626	-
	*Cucumis sativus* var. *sativus*	B10	v3	16 104	(11)
	*Cucumis sativus* var. *hardwickii*	PI 183967	v1	23 667	(12)
	*Cucumis hystrix*	–	v1	23 864	(13)
Watermelon	*Citrullus lanatus* subsp. *vulgaris*	97103	v2.5	21 917	(19)
	*Citrullus lanatus* subsp. *vulgaris*	Charleston Gray	v2.5	22 764	(20)
	*Citrullus lanatus* subsp. *cordophanus*	cordophanus	v2	21 676	(21)
	*Citrullus mucosospermus*	USVL531-MDR	v1	22 377	-
	*Citrullus amarus*	USVL246-FR2	v1	22 028	-
	*Citrullus colocynthis*	PI 537277	v1	22 723	-
Melon	*Cucumis melo*	DHL92	v4.0	28 299	(14)
	*Cucumis melo* var. *inodorus*	Payzawat	v1	22 924	(16)
	*Cucumis melo* ssp. *agrestis*	IVF77	v1	27 073	(15)
	*Cucumis melo* var. *reticulatus*	Harukei-3	v1.41	33 829	(17)
	*Cucumis melo* var *cantalupensis*	Charmono	v1.1	31 348	(18)
	*Cucumis metuliferus*	PI 482460	v1	29 214	(15)
Cucurbita	*Cucurbita maxima*	Rimu	v1.1	32 076	(22)
	*Cucurbita moschata*	Rifu	v1	32 205	(22)
	*Cucurbita pepo* subsp. *pepo*	MU-CU-16	v1	27 868	(23)
	*Cucurbita argyrosperma* subsp. *argyrosperma*	SMH-JMG-627	v2	27 998	(24)
	*Cucurbita argyrosperma* subsp. *sororia*	–	v1	30 592	(24)
Bitter gourd	*Momordica charantia*	OHB3-1	v2	41 016	(28)
	*Momordica charantia*	TR	v1	28 827	(29)
	*Momordica charantia*	Dali-11	v1	26 427	(29)
Bottle gourd	*Lagenaria siceraria*	USVL1VR-Ls	v1	22 472	(31)
	*Lagenaria siceraria*	Hangzhou Gourd	v1	23 510	(30)
Sponge gourd	*Luffa cylindrica*	–	v1	31 661	(25)
	*Luffa cylindrica*	P93075	v1	27 147	(27)
	*Luffa acutangula*	AG-4	v1	42 211	(26)
Wax gourd	*Benincasa hispida*	B227	v1	27 467	(32)
Chayote	*Sechium edule*	–	v1	28 237	(33)
Monk fruit	*Siraitia grosvenorii*	Qingpiguo	v1	30 565	(34)
Snake gourd	*Trichosanthes anguina*	–	v1	22 874	(35)

Genomic synteny blocks and syntenic gene pairs have been identified between any two and within each of the 34 cucurbit genome assemblies, representing a total of 595 pair-wise genome comparisons. Protein sequences from the genomes were first compared against each other (between two genomes) or against themselves (within each genome) using DIAMOND BLASTP (36) with an E-value cutoff of 1e–10 and a maximum of five alignments. The BLASTP results were then fed to MCScanX (37) to identify synteny blocks with default parameters. In total, 391 379 synteny blocks and 12 130 719 syntenic gene pairs, with an average of 31 gene pairs per synteny block, have been identified for the 34 cucurbit genomes, and are stored in MySQL database tables in CuGenDBv2.

### Cucurbit genes and annotations

A total of 919 903 protein-coding genes predicted from the 34 cucurbit genome assemblies have been comprehensively annotated using various public protein and domain databases. Protein sequences of the protein-coding genes were compared against the GenBank non-redundant (nr) (38), UniProt (SwissProt/TrEMBL) (39) and Arabidopsis (TAIR10) protein databases (40) using DIAMOND BLASTP (36) with parameters ‘–more-sensitive –masking 0 –evalue 1e-4’. The conserved domains or motifs in the protein-coding genes were identified by searching their protein sequences against the 16 member databases in InterPro (41) using InterProScan (42). Gene ontology (GO) (43) terms were assigned to each protein-coding gene with the BLAST2GO program (44) using the DIAMOND BLASTP results against the nr database and the results from InterProScan. The human-readable functional description of each protein-coding gene was derived from the BLASTP results against the SwissProt/TrEMBL and TAIR10 protein databases using the AHRD program (https://github.com/groupschoof/AHRD). The Pathway Tools software (45) was used to predict metabolic pathways from protein-coding genes in each of the 34 cucurbit genomes. All these analysis results were uploaded into the PostgreSQL database tables organized by the Chado schema (46) through the data loader function implemented in Tripal v3.0.

### Cucurbit genome variants

During the past several years, high-density genomic variants such as SNPs and small indels have been identified for cucurbit species through large-scale genome resequencing or genotyping-by-sequencing (GBS). SNPs derived from GBS data of 1365 watermelon (25 308 SNPs) (20), 2083 melon (32 268) (47), 1234 cucumber (18 842) (48), 830 *Cucurbita pepo* (47 544), 372 *C. maxima* (5600) and 314 *C. moschata* (46 924) accessions are currently available in CuGenDBv2. SNPs were called from the GBS reads using the TASSEL 5.0 GBS Discovery Pipeline (49) and only biallelic SNPs with minor allele frequency >0.01 were retained. Recently, based on the GBS SNP data, we have constructed a cucumber core collection comprising 388 accessions and performed genome resequencing (∼30×) for these accessions. For watermelon, we previously reported the genome resequencing of 414 accessions (19). We recently generated additional genome sequencing data for 201 accessions, mainly from the wild progenitor and relatives including *C. lanatus* ssp. *cordophanus*, *C. mucosospermus*, *C. amarus* and *C. colocynthis*. After integrating the two datasets, we obtained a genome-sequenced panel of 547 distinct accessions. These genome resequencing data were first processed to remove adaptor and low-quality sequences using Trimmomatic (50), and the cleaned reads were aligned to the representative cucumber (Gy14 v2.1) and watermelon (97103 v2.5) reference genomes, respectively, with BWA-MEM (51). SNPs and small indels were then called using the Sentieon software package (https://www.sentieon.com/), and same as GBS SNPs, only biallelic SNPs and small indels with minor allele frequency >0.01 were kept. A total of 2 513 882 SNPs and 490 882 small indels were identified for the cucumber core collection, and 13 256 154 SNPs and 2 277 760 small indels for the watermelon resequencing panel. All the SNPs and small indels in CuGenDBv2 were functionally annotated by predicting their effects on protein-coding genes using SnpEff (52). SNPs and small indels are stored in the indexed VCF files in which variants can be quickly explored with BCFtools (53). The metadata associated with SNP and small indel variants such as sample accession information are stored in MySQL database tables of CuGenDBv2.

### Cucurbit gene expression profiles

All raw RNA-Seq data (fastq files) from cucurbit species for which reference genomes are available in CuGenDBv2 have been downloaded from NCBI Sequence Read Archive (SRA), as well as the associated project and sample metadata. The metadata were manually curated by checking the publications describing the data (if available), and one brief and informative description for each sample was derived. RNA-Seq data with ambiguous sample information were not included in CuGenDBv2. Raw RNA-Seq reads were first processed to remove adaptor and low-quality sequences using Trimmomatic (50) and polyA/T tails using PRINSEQ++ (54). The processed reads were then aligned to the rRNA database (55) to remove possible contaminating rRNA reads. The final cleaned reads were aligned to the corresponding reference genomes in CuGenBDv2 using HISAT2 (56). Following alignments, raw counts for each protein-coding gene were calculated and then normalized to fragments per kilobase of transcript per million mapped fragments (FPKM). Currently, a total of 221 projects, 1513 distinct samples and 3560 runs (or libraries) are available in CuGenDBv2 (Table 2). The read processing and alignment statistics, raw counts and expression values (FPKM) for each project are available from the CuGenDBv2 download site. The expression value data and the associated project and sample metadata are stored in MySQL database tables of CuGenDBv2.

**Table 2.** Summary of RNA-Seq gene expression data in CuGenDBv2

Species	No. projects	No. samples	No. runs	Reference genomes used
Cucumber (*Cucumis sativus*)	88	523	1315	Chinese Long v3, Gy14 v2.1, B10 v3, PI183967 v1
Cucumber (*Cucumis hystrix*)	2	9	17	hystrix v1
Watermelon (*Citrullus* spp.)	49	293	769	97103 v2.5, Charleston Gray v2.5, USVL246-FR2 v1, PI 537277 v1, cordophanus v2, USVL531-MDR v1
Melon (*Cucumis melo*)	41	362	705	DHL92 v4, Payzawat v1, IVF77 v1, Harukei-3 v1.41, Charmono v1.1
Melon (*Cucumis metuliferus*)	1	4	4	PI 482460 v1
*Cucurbita pepo*	11	94	203	MU-CU-16 v4.1
*Cucurbita moschata*	15	62	158	Rifu v1
*Cucurbita maxima*	7	24	64	Rimu v1.1
*Cucurbita argyrosperma*	2	10	10	SMH-JMG-627 v2, sororia v1
Bottle gourd (*Lagenaria siceraria*)	6	40	86	Hangzhou Gourd v1, USVL1VR-Ls v1
Bitter gourd (*Momordica charantia*)	4	30	75	OHB3-1 v2, TR v1, Dali-11 v1
Wax gourd (*Benincasa hispida*)	5	28	74	B227 v1
Snake gourd (*Trichosanthes anguina*)	1	7	16	Snake gourd v1
Monk fruit (*Siraitia grosvenorii*)	1	6	15	Qingpiguo v1
Sponge gourd (*Luffa* spp.)	8	21	49	cylindrica v1, AG-4 v1, P93075 v1
Chayote (*Sechium edule*)	0	0	0	Chayote v1
Total	225	1513	3560	-

## DATABASE FUNCTIONS

### Gene interface

**Figure 1.**
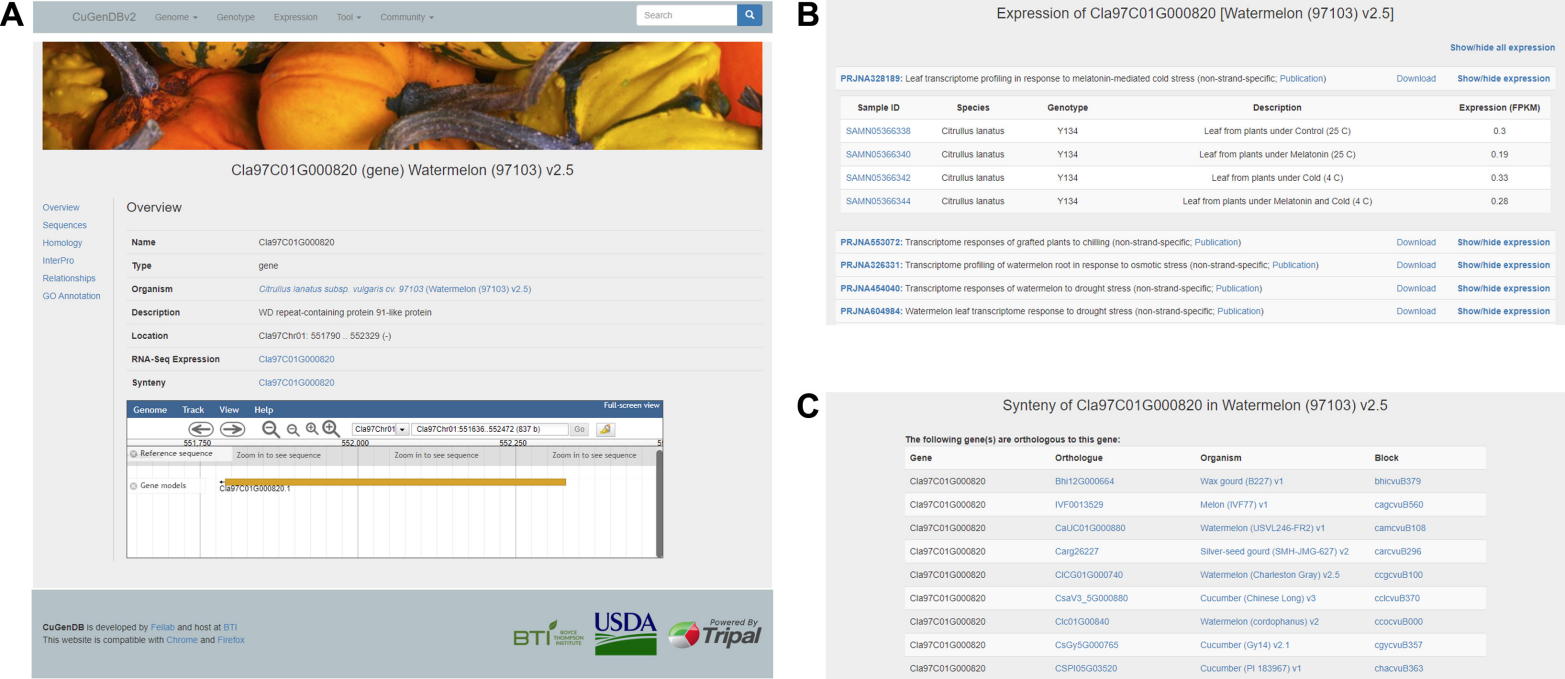
Gene interface in CuGenDBv2. (**A**) Screenshot of the gene feature page showing the navigation menus on the left and gene overview on the right. (**B**) Page showing expression profiles of the queried gene or the gene of interest in different RNA-Seq projects. (**C**) Page showing the list of syntenic orthologous genes of a gene of interest.

Same as in CuGenDBv1, CuGenDBv2 also provides the basic search functions such as search by gene ID or key words, and the batch query function. However, for easier navigation of gene features, the gene page has been redesigned and a navigation bar has been added for different sections related to gene features including ‘Overview’, ‘Sequences’, ‘Homology’, ‘InterPro’, ‘Relationship’ and ‘GO annotation’ (Figure 1A). Besides basic gene features, the ‘Overview’ section also provides links of ‘RNA-Seq Expression’ and ‘Synteny’ for each protein-coding gene. The ‘RNA-Seq Expression’ link displays the expression profiles of the gene of interest in various RNA-Seq projects archived in the database (Figure 1B). The ‘Synteny’ link displays the orthologous and paralogous genes of the gene of interest in different synteny blocks, and the list of synteny blocks that cover the gene (Figure 1C).

### Genotype module

**Figure 2.**
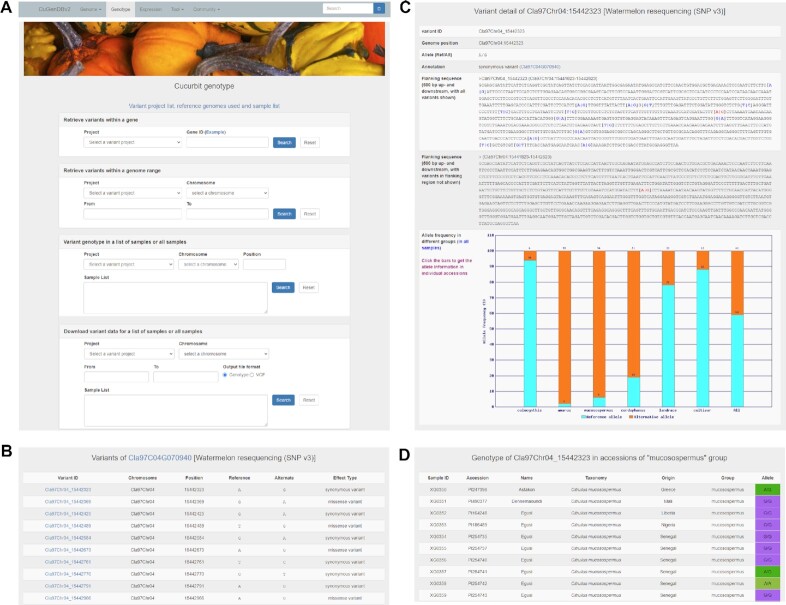
Genotype module in CuGenDBv2. (**A**) Screenshot of the search interfaces of the genotype module. (**B**) Result page showing the list of variants within the genomic region of the queried gene. (**C**) Detailed page of a specific variant. The page shows the basic information of the variant, its genomic flanking sequences, and its allele frequencies in different population groups. (**D**) Detailed genotype information of a specific variant in individual accessions.

A ‘Genotype’ module has been newly implemented in CuGenDBv2 that provides a suite of functions to extract/download variants including SNPs and small indels from large-scale population genome sequencing projects. In this module four variant retrieval/download functions are available: (i) variant retrieval within a gene of interest; (ii) variant retrieval within a specific genomic region; (iii) variant retrieval at a specific genomic position for a list of accessions or all accessions in the project; (iv) download of variant data within a specific genome region for a list of accessions or all accessions in the project (Figure 2A).

Within the genome region of a gene of interest, or a specific genomic region defined by the user, the functions return a list of variants with their genomic positions, the reference and alternate alleles, and their annotations (effects on genes) (Figure 2B). For each specific variant (or variant at a specific genomic position), the interface displays the basic information of the variant, the flanking sequences (500 bp up- and downstream) with or without other variants in the flanking sequences being provided. Moreover, the allele frequencies in different groups from a list of samples or all samples in the project are shown as a bar chart (Figure 2C). Each bar in the chart is linked to a page that displays the genotype information of the variant in individual accessions from the corresponding group (Figure 2D).

### Expression module

To provide a complete cucurbit gene expression atlas, the ‘Expression’ module in CuGenDBv2 has been redesigned. Under this module, the expression profile data of a specific gene can be easily and directly accessed. As described above, expression profiles in different RNA-Seq projects of a gene of interest can be accessed directly through the ‘RNA-Seq Expression’ link provided in the gene page. In addition, the main navigation bar of CuGenDBv2 contains the ‘Expression’ menu, which provides a query interface that also returns the expression profiles (FPKM values) of the queried gene in all corresponding projects/samples. Furthermore, with this redesigned ‘Expression’ module, newly available RNA-Seq expression data can be easily added and displayed in the database.

### Other updated tools

All the other data search, mining and analysis tools in CuGenDBv1 have been preserved in CuGenDBv2. The basic search, analysis and visualization tools, including ‘Search’, ‘BLAST’, ‘JBrowse’, ‘Batch Query’, ‘Synteny Viewer’ and ‘CucurbitCyc’, have been kept with the same functionalities while the backend datasets have been updated with the 34 cucurbit genome assemblies currently available in the database. Specially, synteny blocks between any two and within each of the 34 genome assemblies can be visualized in the database. Functions in the ‘Synteny Viewer’ module have been re-implemented using Perl/CGI with the newly added syntenic genomic data stored in MySQL database tables, which has substantially improved the performance (mainly speed) of these functions. Other data mining and analysis tools, including ‘Pathway enrichment’, ‘GO enrichment’, and ‘Gene classification’, follow the previous designs in CuGenDBv1 with the newly analyzed results from the Pathway Tools and BLAST2GO for the 34 cucurbit genome assemblies.

## CONCLUSIONS AND FUTURE PERSPECTIVES

The CuGenDBv2 currently contains 34 genome assemblies with comprehensive gene functional annotations, from 27 different species/subspecies belonging to 10 cucurbit genera. Compared with CuGenDBv1, a new ‘Genotype’ module has been developed in CuGenDBv2, which helps mining genomic variants including functionally annotated SNPs and small indels identified from large-scale genome sequencing projects with user-friendly interfaces. RNA-Seq raw reads have been downloaded from NCBI SRA for all cucurbit species for which reference genomes are available in CuGenDBv2 and processed to derive gene expression values. The ‘Expression’ module has been redesigned and re-implemented, which provides a complete gene expression atlas for cucurbit species. In addition, CuGenDBv2 includes a huge amount of genomic syntenic information derived from the comparisons of the 34 genomes, and the ‘Synteny Viewer’ have been re-implemented in CuGenDBv2 to improve its performance in handling this type of massive datasets.

CuGenDBv2 will be updated regularly when new genomic datasets are available. New genome assemblies will be included in the database if the assemblies are from species or subspecies that are not covered by the existing genomes in CuGenDBv2 or have substantially higher quality than existing genome assemblies from the same species or subspecies. Variant data can be easily added to the database if the sample metadata are sufficiently clear. Therefore, we will add genome variant data once they are available. RNA-Seq data will be collected from NCBI SRA, processed to derive expression values and included in the database every six months. Furthermore, large-scale phenotypic data are being generated for various cucurbit populations. Functions to mine, analyze and visualize these data and to associate phenotype and genotype data will be implemented in the database.

## DATA AVAILABILITY

All data host in CuGenDBv2 are freely available at (http://cucurbitgenomics.org/v2/).
